# Microstructure, Phase Evolution, and Chemical Behavior of CrCuFeNiTiAlx High Entropy Alloys Processed by Mechanical Alloying

**DOI:** 10.3390/e25020256

**Published:** 2023-01-31

**Authors:** Anay del Ángel-González, Greysi D. Tapía-Higuera, Ibeth Rivera-Ortiz, José A. Castillo-Robles, José A. Rodríguez-García, Carlos A. Calles-Arriaga, José G. Miranda-Hernández, Enrique Rocha-Rangel

**Affiliations:** 1 Departamento de Investigación y Posgrado, Universidad Politécnica de Victoria, Ciudad Victoria 87138, Mexico; 2 Centro Universitario UAEM Valle de México, Laboratorio de Investigación y Desarrollo de Materiales Industriales, Universidad Autónoma del Estado de México, Atizapán de Zaragoza 54500, Mexico

**Keywords:** HEAs, mechanical alloying, phase evolution, chemical properties, FCC-BCC, crystalline structure

## Abstract

High entropy alloys (HEAs) of the type CrCuFeNiTi-Alx were processed through mechanical alloying. The aluminum concentration was varied in the alloy, to determine its effect on the HEAs’ microstructure, phase formation, and chemical behavior. X-ray diffraction studies performed on the pressureless sintered samples revealed the presence of structures composed of face centered cubic (FCC) and body centered cubic (BCC) solid-solution phases. Since the valences of the elements that form the alloy are different, a nearly stoichiometric compound was obtained, increasing the final entropy of the alloy. The aluminum was partly responsible for this situation, which also favored transforming part of the FCC phase into BCC phase on the sintered bodies. X-ray diffraction also indicated the formation of different compounds with the alloy’s metals. Bulk samples exhibited microstructures with different phases. The presence of these phases and the results of the chemical analyses revealed the formation of alloying elements that, in turn, formed a solid solution and, consequently, had a high entropy. From the corrosion tests, it could be concluded that the samples with a lower aluminum content were the most resistant to corrosion.

## 1. Introduction

Metals and their alloys have been part of the solution to the significant challenges faced by humanity since ancient times. These materials generally seek a combination of properties by adding small quantities of one or more metallic element to the main metal. A breakthrough came in 2004 when studies were published on the production of high entropy alloys (HEAs), which represent a new family of metallic materials, opening a new field of study within alloys. High entropy alloys (HEAs) were first described in 2004 [1,2,3] as compounds with at least five metallic elements present in equimolar amounts in the alloy. Despite a large number of alloying elements, HEAs can exhibit simple solid solution phases, such as face centered cubic (FCC); body centered cubic (BCC); and sometimes, depending on the alloying elements, hexagonal phases [4,5,6]. These alloys exhibit a high chemical and thermal stability, which is attributed to the high entropy of mixing associated with a disordered solution of various elements, which is presumed to dominate the enthalpy of phase formation [7,8,9].

High entropy alloys arise from the need to develop new materials for structural applications, where an exceptional mechanical response with properties such as increased strength, good ductility, excellent fracture toughness, good creep, wear resistance, and thermochemical stability are required [10,11]. To date, the most studied system is the one reported in the literature as Cantor alloy, which is composed of CoCrFeMnNi [2]. Recently, the addition of different elements to optimize the microstructure and properties of this system has received attention [12,13,14]. However, the reinforcement mechanisms remain to be clarified [15,16]. A primary characteristic of these studies is that, in many cases, the alloys were manufactured through electric arc furnace melting and casting processes, which leads to the segregation of some elements, such as Cu and Co. HEAs powders can be prepared by mechanical alloying (MA), and then powders can subsequently be sintered to form bulk HEAs, to eliminate casting defects and improve the microstructure. In addition, mechanical alloying also decreases the tendency toward order and leads to an extended solid solubility. Furthermore, the MA route has also been established to facilitate the formation of nanocrystalline HEAs [17]. In this way, metals are processed by mechanical alloying different systems based on CoCrFeMnNi alloy, and other elements such as Al, Mo, Ti, and Zn have been added, seeking to improve the properties of the resultant alloys and trying to explain the mechanism of reinforcement, through observations of the microstructure and measurements of different properties [11,18].

With the development of Ni-based superalloys a few decades ago, a solution to the requirement for structural metals capable of resisting corrosive environments at elevated temperatures was found. Hence, many of the applications of superalloys have been oriented toward the construction of turbines and engine parts. Gas turbines are used to power aircraft, trains, ships, electric generators, and other power generation equipment. Since the working pressures and temperatures must be very high to obtain the best turbine efficiency, materials with a high chemical, thermal, and mechanical stability are needed, to allow them to work under these extreme conditions. Due to their good stability and mechanical properties, high entropy alloys have been proposed for application in the construction of those turbine components that are most exposed to the action of the gases being burned in the turbine. In our group, we have studied the synthesis and mechanical properties of CrCuFeNiTiAlx (where x = 0, 0.5, 1, 2.5, 5 mol %) alloy [19]. In this system, Cr promotes FCC structures, while Cu, Ni, and Ti promote the formation of BCC structures; Ni can help to form both g and g’ phases in superalloys [20,21,22], whereas the mixture of Cr, Ni, and Ti gives corrosion resistance to the alloy [20,21]. Moreover, the addition of Al favors the formation of intermetallic compounds with Fe, Ni, and Ti and also helps to improve corrosion resistance, resulting in lighter alloys [22]. Therefore, the objective of this work was the production through mechanical alloying of various HEAs of the CrCuFeNiTi system, doped with Alx (x = 0, 0.5, 1, 2.5, 5% mol), seeking to determine the effect of aluminum on the microstructure, phase evolution, and chemical behavior of the resulting alloy.

## 2. Materials and Methods

Elemental powders of Al, Cr, Cu, Fe, Ni, and Ti (SkySpring Nanomaterials, Inc., Houston, TX, USA 99.95%, −325 mesh) were mechanically processed with a planetary mill (Retch, PM100, Duesseldorf, Germany), using ZrO_2_ (Tosoh, Grove City, OH, USA) balls of (3 mm in diameter) as the grinding medium and a ball to powder ratio of 12:1 (in weight). Milling was conducted at 300 rpm for 360 min, and 3 mL of isopropyl alcohol was used to control the powder particles’ size distribution and segregation during the ball milling action. The studied system was CrCuFeNiTiAlx, where x = 0, 0.5, 1, 2.5, 5 mol %; the rest of the metals were added in equimolar fractions. Samples were labeled as 1, 2, 3, 4, and 5 for aluminum contents of x = 0, 0.5, 1.0, 2.5, 5.0 mol %, respectively. With the help of a uniaxial press (Montequipo, LAB-30-T, México City, Mexico), the resulting powder from the grinding stage was formed into cylindrical samples of 1.0 by 0.4 cm in diameter and thickness, respectively, using 300 MPa. The pressureless formed samples were sintered in an electrical furnace (Thermo Scientific Thermolyne FB1315M, Waltham, Massachusetts, USA) at 1300 °C for 2 h in an inert atmosphere. Before characterization of the sintered samples, they were ground with SiC sandpaper and then polished using alumina and diamond suspensions. The crystalline phases of the sintered alloys were determined using X-ray diffraction analysis (XRD) with Cu-Kα monochromatic radiation (k = 1.5406 Å) using a step of 0.017 °C and 100 s, performed on X’Pert PRO PANalytical and interpreted with the X’Pert Highscore Plus PANalytical software, using patterns from the ICDD PDF2 database [23]. Next, the obtained microstructure was analyzed using scanning electron microscopy (SEM) and an energy dispersion spectrometer detector (EDS) on a Hitachi SU3500 microscope. The mechanical properties evaluated were as follows: The ultrasonic method determined Young’s modulus, following ASTM standards [24], using Grindosonic A-360 Japanese manufacturing equipment. Microhardness was evaluated in agreement with the ASTM E384–16 standard [25]. In this case, twelve measurements were performed and each of the indentations was made at a spacing distance of at least 5 times the size of each print; the used load was 9.8N for 15 s, and these measurements were performed with a microhardness tester (Wilson Instruments Model S400, USA). The compressive strength was evaluated with a Universal Material Tester WP 300 Gunt. Measurements were taken for 42 days for each of the alloys, which were immersed in a 3% NaCl solution, to simulate an aggressive marine environment, using an Ag/AgCl reference electrode and a Steren multimeter (MUL-005), in order to obtain the corrosion potentials. Linear polarization resistance measurements were performed using a three-electrode array, an Ag/AgCl electrode as a reference, the study sample, and a graphite rod as a counter electrode. Linear polarization resistance was measured according to ASTM G59-97 [26], with operating parameters of ± 15 mV with respect to the corrosion potential (Ecorr), at a sweep rate of 10 mV/min over 42 days.

## 3. Results

### 3.1. Phase Analysis

**Figure 1:**
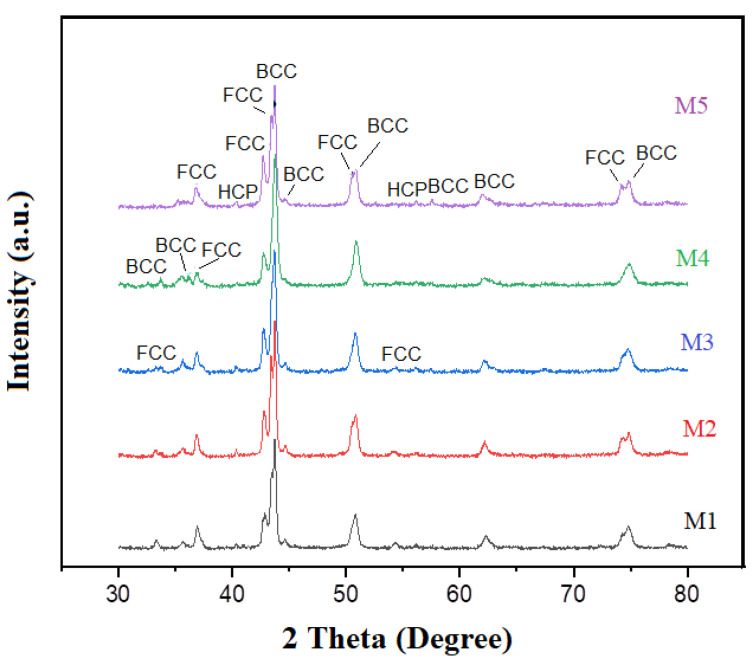
XRD patterns of sintered high entropy CrCuFeNiTiAlx alloys with different Al contents. It is observed that Al-intermetallic and other compounds are formed with Al addition.

The XRD diffractograms presented in Figure 1 indicate that the samples contained very similar phases related to their metallic compounds, intermetallics, and traces of iron and copper oxides. The diffraction patterns showed that solid solutions with a combination of FCC and BCC crystal structures were formed. These crystal structures correspond to those of the alloying metals. However, various compounds formed by the alloying elements were also detected, as depicted in Table 1. The formation of these compounds was due to the type of crystal structure of the interacting element influencing the alloying mechanism, so elements of the same crystal structure were readily dissolved from each other, as was previously documented [27,28]. Previous studies revealed that the alloying order is associated with the melting point of the elements, i.e., the lower the melting point, the easier it is to be alloyed; this is one of the explanations for why aluminum forms several intermetallic compounds [10]. As can be seen in Table 1, Fe, Cr, and Cu oxides were generated during processing. The formation of these oxides is not necessarily negative. Several studies established that configurational disorder can be compositionally engineered into mixed oxides by populating a single sublattice with many distinct cations. The formulations promote novel and entropy-stabilized forms of crystalline matter, where metal cations are incorporated in new ways [29,30]. Finally, Figure 1 shows two small peaks at 40.3 and 56.2 at 2 θ angles, corresponding to a hexagonal close packed (HCP) structure. The presence of this phase was due to the addition of Ti, with Ti being the metal with the highest melting point, only below Cr. However, it is also the metal with the largest atomic radius with an HCP crystalline structure. Both conditions of Ti hindered its solubility in the alloy, hence the two peaks with the HCP phase that appear in the spectra.

### 3.2. Lattice Constant

**Figure 2:**
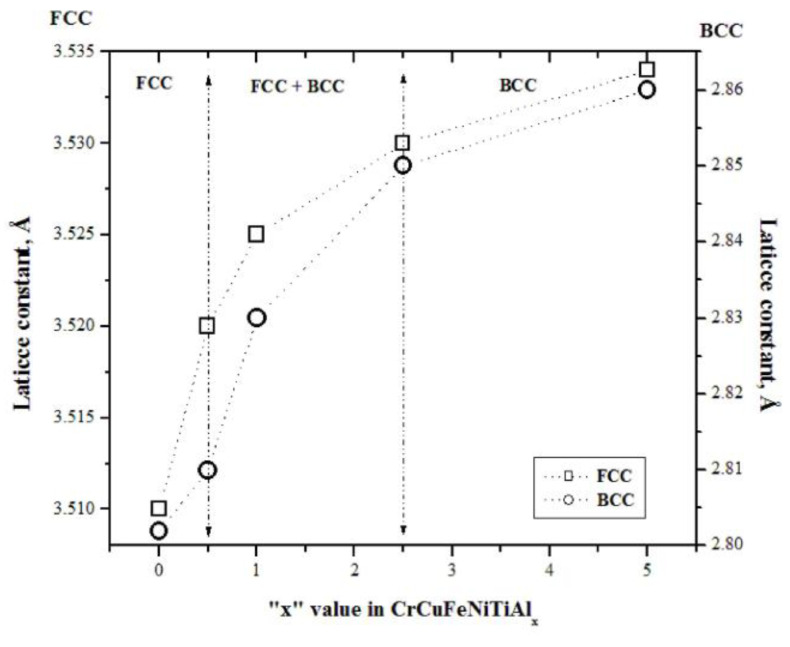
FCC and BCC lattices constants of CrCuFeNiTiAlx alloy, as a function of Al addition.

The lattice constants of the synthesized alloys were calculated using Bragg’s law equation through the interplanar distance (d_hkl_), using the peaks at 2θ = 43.2 and 43.8 °C that correspond to the (111) and (110) planes for the FCC and BCC structures, respectively. Knowing the value of d_hkl_ and using Equation (1) that relates the interplanar distance to the lattice parameter in cubic systems, the latter was determined, and the results are plotted in Figure 2 as a function of the aluminum content in the alloy. In this figure, as the aluminum proportion increases, a considerable increment in the lattice constants of both structures (FCC and BCC) is observed. However, the increment of the lattice constant was more significant for aluminum contents between 0.5 and 2.5% mol in the alloy; for aluminum contents of 5% mol, the increment in lattice constant continued; however, this increment was small. These increases in the lattice constant were associated with the transformation of the FCC crystal structure, first into a mixture of FCC and BCC structures for aluminum contents between 0.5 and 2.5% mol, and then into a dominant BCC structure at a higher aluminum concentration. This phase transformation can be explained as follows: due to its low melting point, aluminum is the first element to be dissolved, diminishing the amount of FCC phase as the aluminum content increases in the alloys. The next metal to dissolve is Cu, which has a smaller atomic radius, further reducing the amount of FCC. Finally, since Cr and Ni are solubilized into the Fe crystalline lattice, there appears to be a greater amount of BCC phase. The latter can be demonstrated by observing the compounds formed and presented in Table 1.

a = d_hkl_(√h^2^ + √k^2^ + √l^2^) (1)

### 3.3. Structural Transformation from FCC to FCC + BCC and BCC

**Figure 3:**
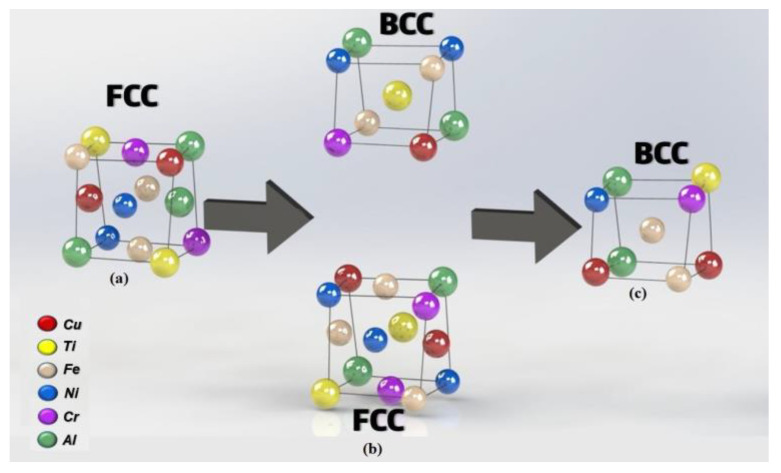
(**a**) Schematic representation of the randomly distributed solid solution structure typical of HEAs made out of Cr, Cu, Fe, Ni, Ti, and Al, which is organized on a distorted FCC crystal structure. (**b**) and (**c**) transformation of FCC to BCC.

As previously discussed, aluminum plays an essential role in the random formation of a solid solution composed of several metals and presenting a mixture of FCC and BCC structure. For the CrCuFeNiTi system studied here, this random solid solution distribution is schematically drawn in Figure 3a, where the Cr, Cu, Fe, Ni, and Ti cations occupy a face-centered cubic (FCC) atomic lattice structure. Since the valence of the cations that form the alloy were different, a nearly stoichiometric compound was obtained, thus increasing the entropy of the alloy. This phase transformation can be explained as follows: due to its low melting point, aluminum is the first element to be dissolved, diminishing the amount of FCC phase as the aluminum content was increased in the alloys. The next metal to dissolve was Cu, which has a smaller atomic radius, further reducing the amount of the FCC. Finally, since Cr and Ni were solubilized into the Fe crystalline lattice, there appears to be a greater amount of BCC phase.

### 3.4. Microstructure and Chemical Analysis

**Figure 4:**
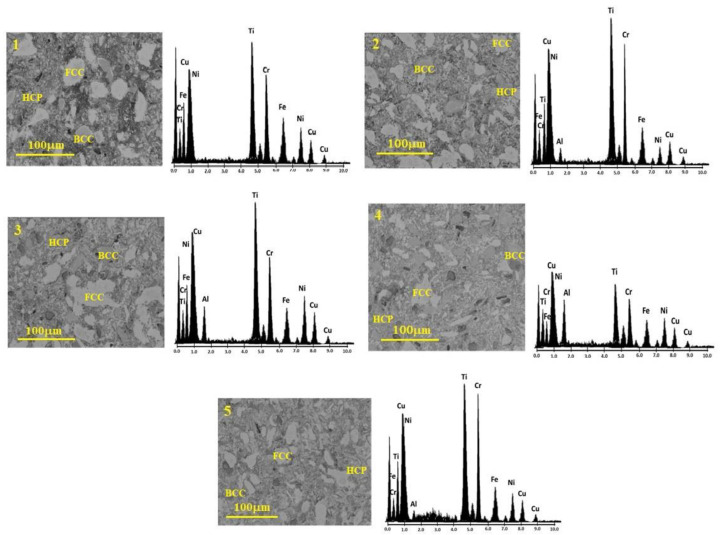
Microstructure and chemical analysis of CrCuFeNiTiAlx alloys.

The microstructure of the various prepared CrCuFeNiTiAlx alloys was characterized by SEM, as shown in Figure 4. In this figure, the microstructures with different phases can be observed and distinguished by their different shades, indicating a variation in the chemical composition of these phases (different phases are labeled in microstructures). Element distribution mapping was performed to corroborate the alloy’s composition. The result of this mapping analysis is shown to the right side of each alloy in Figure 4. The obtained spectrum, which presents the components of the alloy, and a chart with the chemical analysis results, indicate that the resulting chemical composition was close to the hypothesized composition. The presence of these phases in the microstructure and the results of the chemical analyses suggest the formation of alloys between the used elements that, in turn, form a solid solution and, consequently, have a high entropy.

The microstructures in Figure 4 are generally composed of irregular grains of different sizes and morphologies. Bulk samples exhibited particles with multimodal grain sizes, ranging from 0.5 to 50 microns. In the microstructure, larger grains with different contrast (brighter) and characteristics are observed. These bright grains observed at different aluminum concentrations are associated with the presence of the different metallic compounds formed during the processing of the alloys, as was detected by XRD. The greater the amount of Al in the HEAs, the less porosity was observed, i.e., a higher bulk density was achieved. Good densification of the alloys, together with the diversity of phases observed in the microstructure and the fine grain size present, made it possible to obtain alloys with favorable mechanical characteristics.

### 3.5. EDS Mappings

**Figure 5:**
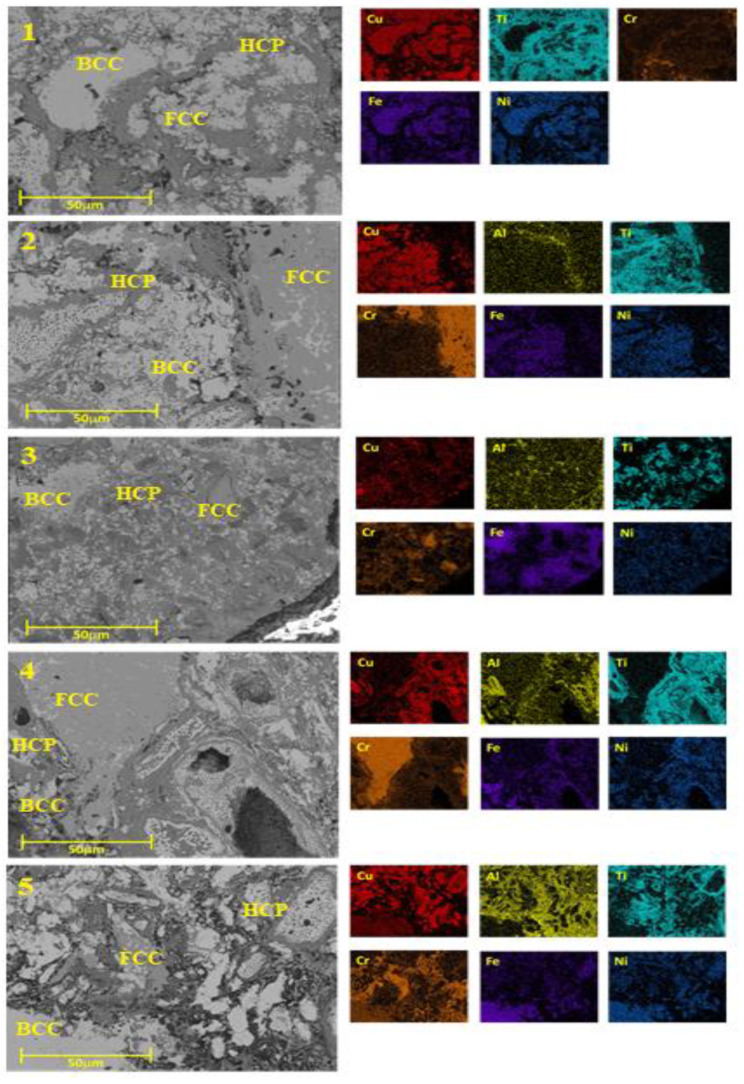
Mappings performed by energy dispersive X-ray spectroscopy of each sample, to verify the spatial distribution of the alloy components.

Through observations and analysis with scanning electron microscopy (SEM) and energy dispersive X-ray spectroscopy (EDS), mapping was performed of each sample to verify the spatial distribution of the alloy components. Figure 5 shows a typical mapping of those performed on the different alloys. The elemental mapping indicated that the samples with sintered CrCuFeNiTiAlx alloys possessed a multi-phase microstructure, as can be seen in the figure by means of the labels placed on the different phases of the microstructure. In the mapping, spatial regions with a good distribution of alloying elements indicated the formation of the high entropy alloys sought here. From these results, it can be inferred that the microstructure of this alloy was composed of a high Cu, Fe, and Ni solid solution (light region); a dark region composed of Ti and Cr; and dispersed chromium and copper oxide phases.

### 3.6. Mechanical Properties

The studied HEAs’ crystalline lattice comprises six metallic elements, each of a different chemical valence and atomic radii. Electrochemical bonding between dissimilar atoms with chemical affinity and atomic size differences undoubtedly leads to lattice distortion [31,32]. The stored strain energy related to the lattice distortion increases the overall free energy of the HEA lattice, thus influencing the physical–chemical properties of the resulting HEAs. In these alloys, Al is present; this is a soft metal with a low melting point, whose addition to HEA alloys leads to their hardening, while diminishing the alloys’ stiffness. Table 2 shows how the microhardness of the sintered CuCrFeNiTiAlx alloys increased, whereas the elastic modulus decreased as a function of the Al content. Evidently, the alloy was hardened significantly by the addition of Al. This result was partly due to the formation of a hard BCC phase and intermetallic compounds of Al with Fe, Ni, and Ti [33]. The results of compressive strength measurements of the manufactured alloys are presented in Table 2. From this table, it can be seen that the sample with 1% Al had the highest compressive strength value. However, the compressive strength decreased considerably for higher aluminum contents (2.5 and 5%), whereas for low values of aluminum (0 or 0.5%), the compressive strength presented acceptable values. These increases in compressive strength may have been due to the nanosized powders obtained from the milling stage (mechanical alloying). According to the Hall–Petch equation, there is an inversely proportional relationship between a material’s strength and grain size, where the reduction in grain size leads to an increase in material strength [30]. On the other hand, Figure 2 shows a transformation of the FCC crystal structure for 0% Al contents to a FCC + BCC mixture for contents of 0.5, 1.0, and 2.5% Al and to BCC for 5% Al contents. At 0.5% Al, the first crystal structure transformation began, obtaining a FCC + BCC mixture, while at 2.5%, this transformation was completed and basically becomes BCC. According to the principal structural factor, a structure with a more slippery system leads to lower lattice friction during dislocation motion and increases the samples’ elasticity [34]. The FCC structure has 48 slip systems, against the BCC structure with 12 slip systems [35]. In this study, the composite contained two FCC phase residues and a single BCC phase. The FCC phases became dominant and hence increased the compression strength and the work hardening effect, and caused the decrease in the stiffness of the alloy observed in Table 2. From all this, it can be said that, because aluminum melts at low temperatures, it generates solution mechanisms, thus greatly contributing to the hardening and reinforcement of the alloy. In conclusion, by increasing the Al concentration, HEA undergoes a microstructural transformation, from a single FCC phase to a coexisting phase mixture of FCC and BCC phases, as well as the precipitation of some intermetallic compounds.

### 3.7. Chemical Behavior

**Figure 6:**
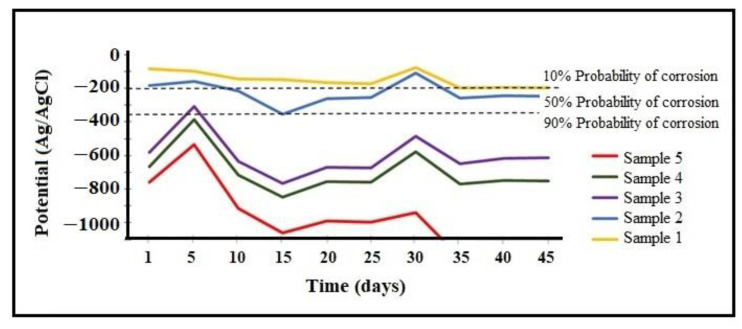
Time-dependent corrosion potential variation of different alloys in 3% NaCl solution.

The variation of corrosion potential over time for working specimens containing the CuCrFeNiAlx alloy (x = 0, 0.5, 1, 2.5, 2.5, 5 mol %) in a 3% NaCl solution can be seen in Figure 6. It is observed that negative corrosion potentials with a 90% probability of corrosion, as per ASTM C876-15 [36], applied to samples 3, 4, and 5, since they are located in the zone with a high probability of corrosion after being submerged for 42 days in 3% NaCl solution. Comparing working samples 1 and 2, we observed that they were initially located in the zone with a 10% probability of corrosion. However, sample 2, 14 days after being submerged in the solution, showed a drop in potential due to the breakage of the passive layer formed by the corrosion products, which placed it in the 50% corrosion probability zone. Sample 1, with a high percentage of copper and titanium and without aluminum, had a lower corrosion rate, which placed it as the specimen with the highest corrosion resistance.

**Figure 7:**
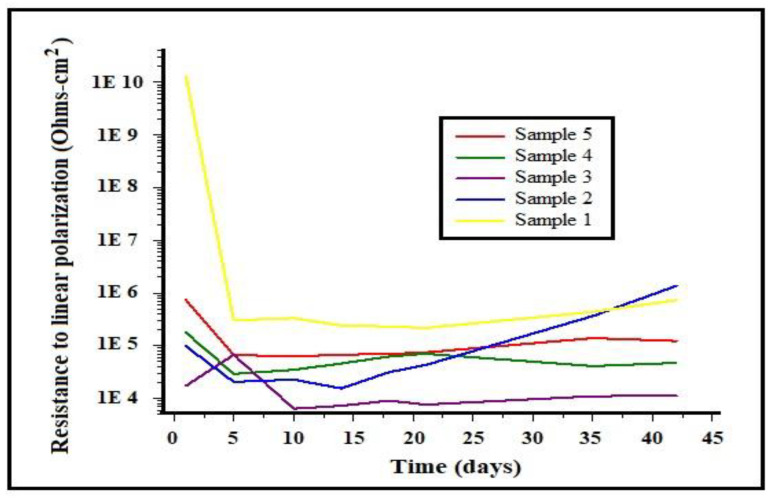
Variation of linear polarization resistance as a function of time for different alloys in 3% NaCl solution.

The linear polarization resistance as a function of time for the different alloys under study in 3% NaCl solution is shown in Figure 7. It can be observed in the graph that samples 3, 4, and 5 showed little resistance, with values between 1 × 10^4^ and 1 × 10^6^ ohms**•**m^2^, in comparison to samples 1 and 2, which showed a greater resistance to the solution, with ascending values between 1 × 10^6^ and 1 × 10^7^ ohms**•**m^2^, and obtaining a better performance. This behavior is in agreement with the results of the corrosion potential measurements. Therefore, it can be concluded that the samples with a lower aluminum content were the most resistant to corrosion.

## 4. Conclusions

Through the proposed methodology, involving high-energy mechanical milling combined with sintering without the application of pressure, it was possible to manufacture high entropy alloys of the CuCrFeNiTiAlx system. Here, the effect of Al on the microstructure, phase evolution, and chemical behavior of the resulting alloys was studied. The following are the main conclusions reached in this study:

The powder metallurgy process was proven as an effective experimental route to retain the characteristic microstructure of high entropy alloys. Since milling induces a finer particle size and high lattice distortion, both conditions facilitate metal bonding in the solid state. Furthermore, sintering promotes metal diffusion and, consequently, their solubility and sample densification.

Based on the XRD studies, as the Al concentration increases, high entropy alloys undergo a microstructural transformation, from a single FCC phase to a mixture of coexisting phases made of FCC and BCC phases, as well as the precipitation of different compounds such as FeNi, Al_3_Ni, CrNiFe, TiAl, and Ti_3_Al. 

The HEA alloy that achieved the highest hardness had the highest Al content. These alloys were hardened significantly with the addition of Al, due to the formation of the BCC phase, the strong atomic bonding between Al and other elements, the larger atomic radius of Al, and the fact that aluminum forms intermetallic compounds, particularly with Ni and Ti.

Samples with a lower aluminum content were the most resistant to corrosion.

## Figures and Tables

**Table 1:** Phases in the diffraction patterns of Figure 1. Sample 4 presented the highest amount of oxidized phases.

Phase	Chemical Formula	Sample				
		1	2	3	4	5
Iron Nickel	Fe_0.64_Ni_0.36_	x	x	x	x	
Iron Nickel	FeNi	x	x	x		x
Awaruite	FeNi					x
Chromium Iron Nickel	CrNiFe	x	x	x		x
Aluminum Nickel	AlNi_3_					x
Copper Nickel	Cu_3.8_Ni	x	x			
Udimet 500	Ni_3_AlTi	x	x	x	x	x
Copper Oxide	Cu_2_O	x	x	x	x	x
Hematite, syn	Fe_2_O_3_	x	x	x		
Chromium oxide	Cr_2_O_3_				x	
Copper Iron Titanium Oxide	Cu_1.2_Ti_0.2_Fe_1.6_O_4_				x	
Copper Iron Titanium	Cu_1.2_Ti_0.2_Fe_1.6_				x	

**Table 2:** Mechanical properties of CuCrFeNiTiAlx alloys.

Sample	Hardness (HV)	Elastic Modulus (GPa)	Compressive Strength (KN/mm^2^)
1	427	167 +/− 10	2211
2	467	161 +/− 9	1777
3	480	153 +/− 9	2393
4	540	142 +/− 10	1973
5	555	137 +/− 9	1043

## Data Availability

In this study new data were not created.
